# *catena*-Poly[[[(5,5,7,12,12,14-hexa­methyl-1,4,8,11-tetra­aza­cyclo­tetra­decane-κ^4^ *N*)nickel(II)]-μ-oxido-[dioxidotungstate(VI)]-μ-oxido] tetra­hydrate]

**DOI:** 10.1107/S1600536812034538

**Published:** 2012-08-11

**Authors:** Guang-Chuan Ou, Xian-You Yuan, Zhi-Zhang Li

**Affiliations:** a Department of Biology and Chemistry, Hunan University of Science and Engineering, Yongzhou Hunan 425100, People’s Republic of China

## Abstract

In the title compound, {[NiWO_4_(C_16_H_36_N_4_)]·4H_2_O}_*n*_, the Ni^II^ ion lies on an inversion center and is octahedrally coordinated by four N atoms of the tetradentate macrocyclic 5,5,7,12,12,14-hexa­methyl-1,4,8,11-tetra­aza­cyclo­tetra­decane (*L*) ligand in the equatorial plane and two O atoms of [WO_4_]^2−^ anions in axial positions. Each [WO_4_]^2−^ anion bridges two adjacent [Ni*L*]^2+^ cations, forming a chain along [001]. The chains are further connected *via* N—H⋯O, O—H⋯O and C—H⋯O hydrogen-bonding inter­actions, generating a three-dimensional structure.

## Related literature

For a related structure, see: Ou *et al.* (2011 ▶).
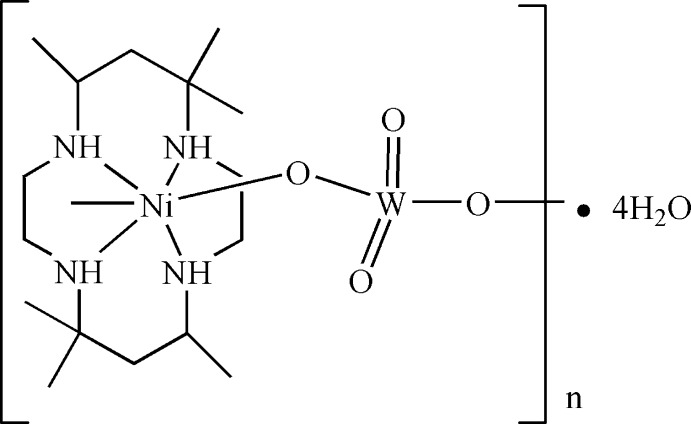


## Experimental

### 

#### Crystal data

[NiWO_4_(C_16_H_36_N_4_)]·4H_2_O
*M* *_r_* = 663.11
Triclinic, 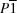

*a* = 8.8402 (14) Å
*b* = 11.7653 (18) Å
*c* = 13.931 (2) Å
α = 107.163 (2)°
β = 102.529 (3)°
γ = 104.984 (3)°
*V* = 1268.1 (3) Å^3^
*Z* = 2
Mo *K*α radiation
μ = 5.32 mm^−1^
*T* = 173 K
0.31 × 0.11 × 0.02 mm

#### Data collection

Bruker SMART CCD area-detector diffractometer
Absorption correction: multi-scan (*SADABS*; Sheldrick, 1996 ▶) *T*_min_ = 0.289, *T*_max_ = 0.901
7631 measured reflections
5397 independent reflections
4330 reflections with *I* > 2σ(*I*)
*R*_int_ = 0.030

#### Refinement

*R*[*F* ^2^ > 2σ(*F* ^2^)] = 0.038
*wR*(*F* ^2^) = 0.096
*S* = 1.02
5397 reflections
304 parameters
13 restraints
H atoms treated by a mixture of independent and constrained refinement
Δρ_max_ = 2.33 e Å^−3^
Δρ_min_ = −1.48 e Å^−3^

### 

Data collection: *SMART* (Bruker, 1999 ▶); cell refinement: *SAINT-Plus* (Bruker, 1999 ▶); data reduction: *SAINT-Plus*; program(s) used to solve structure: *SHELXS97* (Sheldrick, 2008 ▶); program(s) used to refine structure: *SHELXL97* (Sheldrick, 2008 ▶); molecular graphics: *SHELXTL* (Sheldrick, 2008 ▶); software used to prepare material for publication: *SHELXTL*.

## Supplementary Material

Crystal structure: contains datablock(s) I, global. DOI: 10.1107/S1600536812034538/pv2574sup1.cif

Structure factors: contains datablock(s) I. DOI: 10.1107/S1600536812034538/pv2574Isup2.hkl

Additional supplementary materials:  crystallographic information; 3D view; checkCIF report

## Figures and Tables

**Table 1:** Hydrogen-bond geometry (Å, °)

*D*—H⋯*A*	*D*—H	H⋯*A*	*D*⋯*A*	*D*—H⋯*A*
N1—H1*C*⋯O2^i^	0.93	2.32	3.253 (7)	180
N2—H2*C*⋯O4*W* ^i^	0.93	2.21	3.040 (8)	149
O4*W*—H4*WB*⋯O3*W*	0.85 (2)	2.12 (5)	2.720 (8)	128 (6)
O4*W*—H4*WA*⋯O3	0.86 (2)	2.05 (3)	2.900 (7)	168 (6)
O2*W*—H2*WA*⋯O1*W* ^ii^	0.85 (2)	1.94 (2)	2.790 (8)	175 (7)
O3*W*—H3*WA*⋯O2^iii^	0.87 (2)	2.01 (5)	2.784 (7)	148 (8)
O1*W*—H1*WA*⋯O2	0.86 (2)	1.95 (2)	2.801 (7)	172 (8)
O2*W*—H2*WB*⋯O3	0.86 (2)	1.99 (3)	2.811 (7)	160 (8)
O3*W*—H3*WB*⋯O2*W*	0.86 (2)	2.08 (4)	2.834 (8)	145 (6)
O1*W*—H1*WB*⋯O2*W*	0.85 (2)	2.10 (4)	2.895 (9)	157 (7)
C16—H16*A*⋯O1^iv^	0.98	2.40	3.241 (9)	144

Symmetry codes: (i) 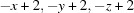; (ii) 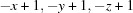; (iii) 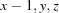; (iv) 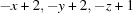.

**Fig. 1.**
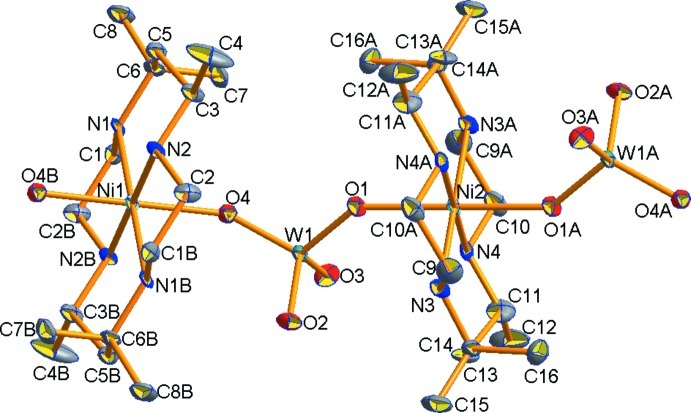
The molecular structure of the title complex, with atom labels and 50% probability displacement ellipsoids for non-H atoms. Symmetry codes for the generated atoms: A: 2 - x, 2 - y, 1 - z; B: 2 - x, 2 - y, 2 - z.

**Fig. 2.**
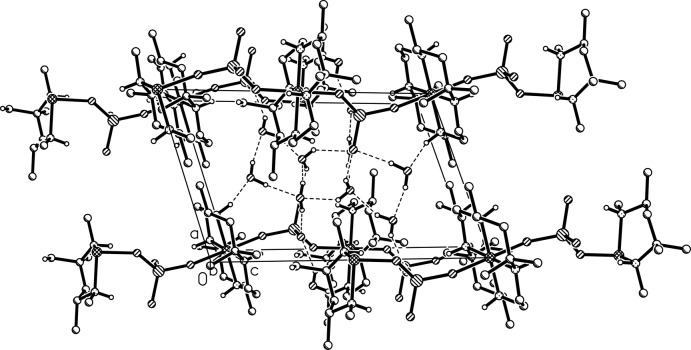
A view of the hydrogen bonds (dotted lines) in the crystal structure of the title compound. H atoms non-participating in hydrogen- bonding were omitted for clarity.

**Fig. 3.**
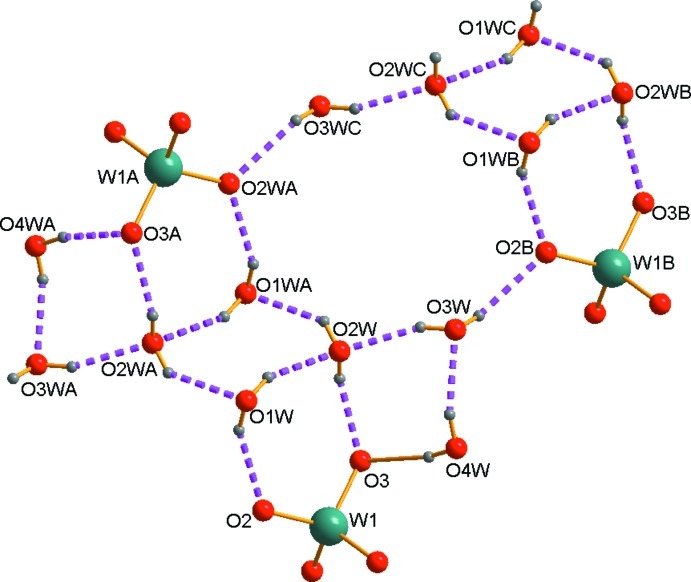
Hydrogen bonding (dashed lines) in the title compound. Symmetry codes for the generated atoms: A: 1 - x, 1 - y, 1 - z; B: -1 + x, y, z; C: - x, 1 - y, 1 - z.

## supplementary crystallographic information

### Comment

Continuing our research (Ou *et al.*, 2011), we now report the crystal structure of the title complex. The asymmetric unit of the title complex contains one cation [NiL]^2+^, one anion [WO_4_]^2-^, and four water molecules of hydration. Each Ni^II^ ion displays a distorted octahedral coordination geometry by coordination with four nitrogen atoms of *L* in in the equatorial plane, and two oxygen atoms of [WO_4_]^2-^ anions in the axial positions. Each [WO_4_]^2-^ anion bridges two adjacent [NiL]^2+ ^cations to form a one-dimensional chain. The one-dimensional chains are further connected through O···O (2.720 (8)–2.900 (8) Å) and N···O (3.040 (8) and 3.253 (7) Å) hydrogen bonding interactions between the oxygen atoms of [WO_4_]^2-^ anions, free water molecules and the secondary amine of [NiL]^2+^, forming a three-dimensional supramolecular structure (Tab. 2, Figs. 2, 3).

### Experimental

A glass tube was charged with an aqueous solution of K_2_WO_4_ (0.033 g, 0.1 mmol) in water (20 ml), and a mixture of methanol and H_2_O (1/1, 20 ml) was gently added as an upper layer. A solution of NiL (ClO_4_)_2_ (0.054 g, 0.1 mmol) (*L* = 5,5,7,12,12,14-hexamethyl-1,4,8,11-tetraazacyclotetradecane) in methanol (20 ml) was added carefully as a third layer, and then the tube was sealed. After several weeks, yellow prism-shaped crystals were obtained.

### Refinement

The H atoms bound to N and C atoms were positioned geometrically and refined using the riding model with N—H = 0.93 Å and C—H = 0.98 to 1.00 Å. The hydrogen atoms of the water molecules were located from a difference Fourier map and were constrained at distances O—H = 0.86 (2) Å. *U*_iso_(H) were set to 1.5 × *U*_eq_(methyl C) and 1.2 × *U*_eq_(the rest of the parent atoms).

### Crystal data

**Table:**

[NiWO_4_(C_16_H_36_N_4_)]·4H_2_O	*Z* = 2
*M**_r_* = 663.11	*F*(000) = 668
Triclinic, *P*1	*D*_x_ = 1.737 Mg m^−^^3^
Hall symbol: -P 1	Mo *K*α radiation, λ = 0.71073 Å
*a* = 8.8402 (14) Å	Cell parameters from 3766 reflections
*b* = 11.7653 (18) Å	θ = 1.9–27.1°
*c* = 13.931 (2) Å	µ = 5.32 mm^−^^1^
α = 107.163 (2)°	*T* = 173 K
β = 102.529 (3)°	Prism, yellow
γ = 104.984 (3)°	0.31 × 0.11 × 0.02 mm
*V* = 1268.1 (3) Å^3^	

### Data collection

**Table:**

Bruker SMART CCD area-detector diffractometer	5397 independent reflections
Radiation source: fine-focus sealed tube	4330 reflections with *I* > 2σ(*I*)
Graphite monochromator	*R*_int_ = 0.030
φ and ω scans	θ_max_ = 27.1°, θ_min_ = 1.9°
Absorption correction: multi-scan (*SADABS*; Sheldrick, 1996)	*h* = −11→4
*T*_min_ = 0.289, *T*_max_ = 0.901	*k* = −14→15
7631 measured reflections	*l* = −17→17

### Refinement

**Table:**

Refinement on *F*^2^	Primary atom site location: structure-invariant direct methods
Least-squares matrix: full	Secondary atom site location: difference Fourier map
*R*[*F*^2^ > 2σ(*F*^2^)] = 0.038	Hydrogen site location: inferred from neighbouring sites
*wR*(*F*^2^) = 0.096	H atoms treated by a mixture of independent and constrained refinement
*S* = 1.02	*w* = 1/[σ^2^(*F*_o_^2^) + (0.0498*P*)^2^] where *P* = (*F*_o_^2^ + 2*F*_c_^2^)/3
5397 reflections	(Δ/σ)_max_ = 0.001
304 parameters	Δρ_max_ = 2.33 e Å^−^^3^
13 restraints	Δρ_min_ = −1.48 e Å^−^^3^

### Special details

**Table:**

Geometry. All e.s.d.'s (except the e.s.d. in the dihedral angle between two l.s. planes) are estimated using the full covariance matrix. The cell e.s.d.'s are taken into account individually in the estimation of e.s.d.'s in distances, angles and torsion angles; correlations between e.s.d.'s in cell parameters are only used when they are defined by crystal symmetry. An approximate (isotropic) treatment of cell e.s.d.'s is used for estimating e.s.d.'s involving l.s. planes.
Refinement. Refinement of *F*^2^ against ALL reflections. The weighted *R*-factor *wR* and goodness of fit *S* are based on *F*^2^, conventional *R*-factors *R* are based on *F*, with *F* set to zero for negative *F*^2^. The threshold expression of *F*^2^ > σ(*F*^2^) is used only for calculating *R*-factors(gt) *etc*. and is not relevant to the choice of reflections for refinement. *R*-factors based on *F*^2^ are statistically about twice as large as those based on *F*, and *R*- factors based on ALL data will be even larger.

### Fractional atomic coordinates and isotropic or equivalent isotropic displacement parameters (Å^2^)

**Table:**

	*x*	*y*	*z*	*U*_iso_*/*U*_eq_	
W1	0.86432 (3)	0.90496 (2)	0.70182 (2)	0.01423 (9)	
O4	0.9085 (5)	0.9844 (4)	0.8399 (3)	0.0187 (9)	
O1	0.9900 (5)	0.9988 (4)	0.6505 (3)	0.0201 (10)	
O3	0.6535 (5)	0.8719 (4)	0.6342 (4)	0.0231 (10)	
O2	0.9049 (6)	0.7611 (4)	0.6806 (4)	0.0258 (11)	
Ni1	1.0000	1.0000	1.0000	0.0130 (2)	
Ni2	1.0000	1.0000	0.5000	0.0139 (2)	
N1	0.9427 (7)	1.1622 (5)	1.0653 (4)	0.0170 (11)	
H1C	0.9860	1.1842	1.1380	0.020*	
N2	1.2351 (6)	1.1000 (5)	1.0097 (4)	0.0158 (11)	
H2C	1.3028	1.1227	1.0785	0.019*	
N4	0.7426 (6)	0.9212 (5)	0.4386 (4)	0.0183 (11)	
H4D	0.7043	0.9048	0.4920	0.022*	
N3	1.0343 (7)	0.8257 (5)	0.4733 (4)	0.0195 (12)	
H3A	1.0258	0.8088	0.5334	0.023*	
C6	1.0127 (9)	1.2828 (6)	1.0490 (5)	0.0219 (15)	
C11	0.6783 (9)	0.7997 (7)	0.3458 (6)	0.0298 (17)	
H11	0.7226	0.8148	0.2887	0.036*	
C5	1.1979 (9)	1.3084 (6)	1.0670 (6)	0.0264 (16)	
H5A	1.2466	1.3084	1.1380	0.032*	
H5B	1.2477	1.3953	1.0694	0.032*	
C9	1.2125 (8)	0.8541 (7)	0.4833 (6)	0.0271 (16)	
H9A	1.2321	0.8652	0.4188	0.033*	
H9B	1.2474	0.7833	0.4922	0.033*	
C10	0.6896 (9)	1.0242 (7)	0.4200 (6)	0.0295 (17)	
H10A	0.5698	1.0036	0.4095	0.035*	
H10B	0.7109	1.0349	0.3558	0.035*	
C14	0.9159 (9)	0.7086 (6)	0.3836 (6)	0.0266 (16)	
C16	0.9464 (10)	0.7001 (7)	0.2778 (6)	0.0359 (19)	
H16A	0.9446	0.7773	0.2649	0.054*	
H16B	0.8597	0.6263	0.2204	0.054*	
H16C	1.0543	0.6912	0.2805	0.054*	
C13	0.7386 (9)	0.7023 (6)	0.3763 (6)	0.0313 (18)	
H13A	0.6640	0.6175	0.3245	0.038*	
H13B	0.7252	0.7078	0.4461	0.038*	
C15	0.9397 (11)	0.5905 (7)	0.4055 (7)	0.041 (2)	
H15A	0.8627	0.5129	0.3469	0.061*	
H15B	0.9180	0.5911	0.4717	0.061*	
H15C	1.0533	0.5934	0.4117	0.061*	
C12	0.4873 (10)	0.7496 (8)	0.3017 (7)	0.045 (2)	
H12A	0.4416	0.7344	0.3568	0.068*	
H12B	0.4495	0.6701	0.2403	0.068*	
H12C	0.4499	0.8125	0.2803	0.068*	
C3	1.2531 (9)	1.2195 (6)	0.9881 (6)	0.0256 (16)	
H3	1.1785	1.1969	0.9150	0.031*	
C8	0.9959 (10)	1.3946 (6)	1.1322 (6)	0.0283 (16)	
H8A	0.8787	1.3813	1.1227	0.042*	
H8B	1.0477	1.4736	1.1233	0.042*	
H8C	1.0506	1.4000	1.2034	0.042*	
C7	0.9231 (9)	1.2735 (7)	0.9384 (6)	0.0298 (17)	
H7A	0.9179	1.1950	0.8854	0.045*	
H7B	0.9832	1.3464	0.9246	0.045*	
H7C	0.8109	1.2731	0.9343	0.045*	
C4	1.4286 (10)	1.2865 (8)	0.9937 (8)	0.051 (3)	
H4A	1.5007	1.3220	1.0674	0.077*	
H4B	1.4304	1.3550	0.9671	0.077*	
H4C	1.4677	1.2257	0.9504	0.077*	
C1	0.7623 (8)	1.1146 (6)	1.0442 (5)	0.0213 (14)	
H1A	0.7047	1.1009	0.9702	0.026*	
H1B	0.7301	1.1780	1.0919	0.026*	
C2	1.2872 (8)	1.0076 (6)	0.9384 (5)	0.0188 (14)	
H2A	1.4084	1.0407	0.9539	0.023*	
H2B	1.2336	0.9936	0.8636	0.023*	
O2W	0.3988 (7)	0.6388 (5)	0.5720 (5)	0.0422 (14)	
H2WA	0.389 (10)	0.589 (5)	0.510 (3)	0.051*	
H2WB	0.458 (9)	0.714 (3)	0.582 (5)	0.051*	
O4W	0.5200 (7)	0.9166 (6)	0.8089 (4)	0.0441 (15)	
H4WB	0.461 (7)	0.842 (3)	0.797 (4)	0.053*	
H4WA	0.564 (9)	0.915 (6)	0.759 (5)	0.053*	
O1W	0.6501 (8)	0.5326 (5)	0.6291 (5)	0.0447 (15)	
H1WA	0.729 (5)	0.599 (5)	0.639 (7)	0.054*	
H1WB	0.560 (4)	0.547 (7)	0.617 (7)	0.054*	
O3W	0.2150 (7)	0.7401 (6)	0.6956 (5)	0.0504 (17)	
H3WA	0.109 (3)	0.714 (8)	0.681 (5)	0.061*	
H3WB	0.234 (7)	0.706 (8)	0.638 (3)	0.061*	

### Atomic displacement parameters (Å^2^)

**Table:**

	*U*^11^	*U*^22^	*U*^33^	*U*^12^	*U*^13^	*U*^23^
W1	0.01641 (14)	0.01438 (14)	0.01496 (14)	0.00471 (10)	0.00913 (10)	0.00725 (10)
O4	0.021 (2)	0.019 (2)	0.017 (2)	0.0070 (19)	0.0074 (19)	0.0073 (19)
O1	0.014 (2)	0.023 (2)	0.021 (3)	0.0006 (19)	0.0067 (19)	0.010 (2)
O3	0.015 (2)	0.028 (3)	0.022 (3)	0.003 (2)	0.007 (2)	0.004 (2)
O2	0.029 (3)	0.018 (2)	0.034 (3)	0.012 (2)	0.010 (2)	0.011 (2)
Ni1	0.0154 (6)	0.0136 (5)	0.0142 (6)	0.0060 (4)	0.0092 (5)	0.0070 (4)
Ni2	0.0161 (6)	0.0127 (5)	0.0161 (6)	0.0038 (4)	0.0092 (5)	0.0077 (4)
N1	0.027 (3)	0.016 (3)	0.019 (3)	0.011 (2)	0.016 (2)	0.012 (2)
N2	0.020 (3)	0.016 (3)	0.017 (3)	0.008 (2)	0.009 (2)	0.011 (2)
N4	0.019 (3)	0.021 (3)	0.018 (3)	0.006 (2)	0.008 (2)	0.010 (2)
N3	0.023 (3)	0.021 (3)	0.021 (3)	0.010 (2)	0.010 (2)	0.012 (2)
C6	0.039 (4)	0.015 (3)	0.021 (4)	0.016 (3)	0.017 (3)	0.010 (3)
C11	0.020 (4)	0.031 (4)	0.029 (4)	−0.001 (3)	0.007 (3)	0.008 (3)
C5	0.036 (4)	0.016 (3)	0.032 (4)	0.008 (3)	0.022 (3)	0.010 (3)
C9	0.024 (4)	0.038 (4)	0.030 (4)	0.020 (3)	0.013 (3)	0.017 (3)
C10	0.026 (4)	0.032 (4)	0.045 (5)	0.015 (3)	0.019 (4)	0.023 (4)
C14	0.042 (5)	0.015 (3)	0.024 (4)	0.007 (3)	0.020 (3)	0.007 (3)
C16	0.047 (5)	0.029 (4)	0.028 (4)	0.010 (4)	0.018 (4)	0.005 (3)
C13	0.042 (5)	0.013 (3)	0.029 (4)	−0.004 (3)	0.012 (4)	0.004 (3)
C15	0.059 (6)	0.018 (4)	0.046 (5)	0.013 (4)	0.024 (4)	0.010 (4)
C12	0.038 (5)	0.032 (5)	0.045 (5)	0.002 (4)	0.001 (4)	0.004 (4)
C3	0.028 (4)	0.017 (3)	0.041 (5)	0.009 (3)	0.024 (3)	0.015 (3)
C8	0.044 (5)	0.019 (4)	0.032 (4)	0.017 (3)	0.021 (4)	0.011 (3)
C7	0.044 (5)	0.032 (4)	0.027 (4)	0.021 (4)	0.016 (4)	0.020 (3)
C4	0.039 (5)	0.029 (5)	0.098 (8)	0.008 (4)	0.041 (5)	0.032 (5)
C1	0.024 (4)	0.021 (3)	0.025 (4)	0.012 (3)	0.013 (3)	0.010 (3)
C2	0.016 (3)	0.019 (3)	0.025 (4)	0.007 (3)	0.015 (3)	0.006 (3)
O2W	0.031 (3)	0.029 (3)	0.055 (4)	0.001 (3)	0.020 (3)	0.003 (3)
O4W	0.027 (3)	0.071 (4)	0.029 (3)	0.012 (3)	0.015 (3)	0.012 (3)
O1W	0.041 (3)	0.027 (3)	0.048 (4)	−0.003 (3)	0.000 (3)	0.011 (3)
O3W	0.032 (3)	0.044 (4)	0.060 (4)	0.007 (3)	0.025 (3)	−0.005 (3)

### Geometric parameters (Å, º)

**Table:**

W1—O1	1.772 (4)	C10—H10A	0.9900
W1—O2	1.773 (4)	C10—H10B	0.9900
W1—O4	1.775 (4)	C14—C13	1.529 (10)
W1—O3	1.778 (4)	C14—C16	1.534 (9)
O4—Ni1	2.135 (4)	C14—C15	1.563 (9)
O1—Ni2	2.121 (4)	C16—H16A	0.9800
Ni1—N2	2.059 (5)	C16—H16B	0.9800
Ni1—N2^i^	2.059 (5)	C16—H16C	0.9800
Ni1—N1^i^	2.098 (5)	C13—H13A	0.9900
Ni1—N1	2.098 (5)	C13—H13B	0.9900
Ni1—O4^i^	2.135 (4)	C15—H15A	0.9800
Ni2—N3^ii^	2.087 (5)	C15—H15B	0.9800
Ni2—N3	2.087 (5)	C15—H15C	0.9800
Ni2—N4^ii^	2.089 (5)	C12—H12A	0.9800
Ni2—N4	2.089 (5)	C12—H12B	0.9800
Ni2—O1^ii^	2.121 (4)	C12—H12C	0.9800
N1—C1	1.477 (8)	C3—C4	1.521 (10)
N1—C6	1.498 (8)	C3—H3	1.0000
N1—H1C	0.9300	C8—H8A	0.9800
N2—C2	1.484 (7)	C8—H8B	0.9800
N2—C3	1.499 (8)	C8—H8C	0.9800
N2—H2C	0.9300	C7—H7A	0.9800
N4—C10	1.478 (8)	C7—H7B	0.9800
N4—C11	1.486 (8)	C7—H7C	0.9800
N4—H4D	0.9300	C4—H4A	0.9800
N3—C9	1.490 (8)	C4—H4B	0.9800
N3—C14	1.492 (8)	C4—H4C	0.9800
N3—H3A	0.9300	C1—C2^i^	1.498 (8)
C6—C7	1.530 (9)	C1—H1A	0.9900
C6—C5	1.535 (10)	C1—H1B	0.9900
C6—C8	1.539 (9)	C2—C1^i^	1.498 (8)
C11—C13	1.511 (10)	C2—H2A	0.9900
C11—C12	1.548 (10)	C2—H2B	0.9900
C11—H11	1.0000	O2W—H2WA	0.85 (2)
C5—C3	1.536 (9)	O2W—H2WB	0.86 (2)
C5—H5A	0.9900	O4W—H4WB	0.845 (19)
C5—H5B	0.9900	O4W—H4WA	0.86 (2)
C9—C10^ii^	1.526 (10)	O1W—H1WA	0.86 (2)
C9—H9A	0.9900	O1W—H1WB	0.85 (2)
C9—H9B	0.9900	O3W—H3WA	0.87 (2)
C10—C9^ii^	1.526 (10)	O3W—H3WB	0.863 (19)
			
O1—W1—O2	108.7 (2)	N3—C9—H9A	110.2
O1—W1—O4	110.9 (2)	C10^ii^—C9—H9A	110.2
O2—W1—O4	108.7 (2)	N3—C9—H9B	110.2
O1—W1—O3	108.9 (2)	C10^ii^—C9—H9B	110.2
O2—W1—O3	109.7 (2)	H9A—C9—H9B	108.5
O4—W1—O3	109.9 (2)	N4—C10—C9^ii^	108.0 (6)
W1—O4—Ni1	151.1 (2)	N4—C10—H10A	110.1
W1—O1—Ni2	137.9 (2)	C9^ii^—C10—H10A	110.1
N2—Ni1—N2^i^	180.000 (1)	N4—C10—H10B	110.1
N2—Ni1—N1^i^	85.46 (19)	C9^ii^—C10—H10B	110.1
N2^i^—Ni1—N1^i^	94.5 (2)	H10A—C10—H10B	108.4
N2—Ni1—N1	94.5 (2)	N3—C14—C13	109.8 (5)
N2^i^—Ni1—N1	85.46 (19)	N3—C14—C16	112.3 (6)
N1^i^—Ni1—N1	180.000 (3)	C13—C14—C16	110.8 (6)
N2—Ni1—O4	90.57 (18)	N3—C14—C15	108.7 (6)
N2^i^—Ni1—O4	89.43 (18)	C13—C14—C15	108.1 (6)
N1^i^—Ni1—O4	85.20 (18)	C16—C14—C15	106.9 (6)
N1—Ni1—O4	94.80 (18)	C14—C16—H16A	109.5
N2—Ni1—O4^i^	89.43 (18)	C14—C16—H16B	109.5
N2^i^—Ni1—O4^i^	90.57 (18)	H16A—C16—H16B	109.5
N1^i^—Ni1—O4^i^	94.80 (18)	C14—C16—H16C	109.5
N1—Ni1—O4^i^	85.20 (18)	H16A—C16—H16C	109.5
O4—Ni1—O4^i^	180.000 (2)	H16B—C16—H16C	109.5
N3^ii^—Ni2—N3	180.000 (2)	C11—C13—C14	118.9 (6)
N3^ii^—Ni2—N4^ii^	94.6 (2)	C11—C13—H13A	107.6
N3—Ni2—N4^ii^	85.4 (2)	C14—C13—H13A	107.6
N3^ii^—Ni2—N4	85.4 (2)	C11—C13—H13B	107.6
N3—Ni2—N4	94.6 (2)	C14—C13—H13B	107.6
N4^ii^—Ni2—N4	180.000 (1)	H13A—C13—H13B	107.0
N3^ii^—Ni2—O1^ii^	86.13 (19)	C14—C15—H15A	109.5
N3—Ni2—O1^ii^	93.87 (19)	C14—C15—H15B	109.5
N4^ii^—Ni2—O1^ii^	90.23 (18)	H15A—C15—H15B	109.5
N4—Ni2—O1^ii^	89.77 (18)	C14—C15—H15C	109.5
N3^ii^—Ni2—O1	93.87 (19)	H15A—C15—H15C	109.5
N3—Ni2—O1	86.13 (19)	H15B—C15—H15C	109.5
N4^ii^—Ni2—O1	89.77 (18)	C11—C12—H12A	109.5
N4—Ni2—O1	90.23 (18)	C11—C12—H12B	109.5
O1^ii^—Ni2—O1	180.000 (2)	H12A—C12—H12B	109.5
C1—N1—C6	116.7 (5)	C11—C12—H12C	109.5
C1—N1—Ni1	104.6 (4)	H12A—C12—H12C	109.5
C6—N1—Ni1	122.1 (4)	H12B—C12—H12C	109.5
C1—N1—H1C	103.7	N2—C3—C4	112.5 (6)
C6—N1—H1C	103.7	N2—C3—C5	109.4 (5)
Ni1—N1—H1C	103.7	C4—C3—C5	110.3 (6)
C2—N2—C3	113.8 (5)	N2—C3—H3	108.2
C2—N2—Ni1	105.4 (4)	C4—C3—H3	108.2
C3—N2—Ni1	115.3 (4)	C5—C3—H3	108.2
C2—N2—H2C	107.3	C6—C8—H8A	109.5
C3—N2—H2C	107.3	C6—C8—H8B	109.5
Ni1—N2—H2C	107.3	H8A—C8—H8B	109.5
C10—N4—C11	115.2 (5)	C6—C8—H8C	109.5
C10—N4—Ni2	104.7 (4)	H8A—C8—H8C	109.5
C11—N4—Ni2	114.5 (4)	H8B—C8—H8C	109.5
C10—N4—H4D	107.3	C6—C7—H7A	109.5
C11—N4—H4D	107.3	C6—C7—H7B	109.5
Ni2—N4—H4D	107.3	H7A—C7—H7B	109.5
C9—N3—C14	116.5 (5)	C6—C7—H7C	109.5
C9—N3—Ni2	104.7 (4)	H7A—C7—H7C	109.5
C14—N3—Ni2	121.3 (4)	H7B—C7—H7C	109.5
C9—N3—H3A	104.1	C3—C4—H4A	109.5
C14—N3—H3A	104.1	C3—C4—H4B	109.5
Ni2—N3—H3A	104.1	H4A—C4—H4B	109.5
N1—C6—C7	110.9 (6)	C3—C4—H4C	109.5
N1—C6—C5	107.7 (5)	H4A—C4—H4C	109.5
C7—C6—C5	111.8 (6)	H4B—C4—H4C	109.5
N1—C6—C8	110.2 (5)	N1—C1—C2^i^	109.7 (5)
C7—C6—C8	108.9 (5)	N1—C1—H1A	109.7
C5—C6—C8	107.4 (6)	C2^i^—C1—H1A	109.7
N4—C11—C13	109.7 (6)	N1—C1—H1B	109.7
N4—C11—C12	111.8 (6)	C2^i^—C1—H1B	109.7
C13—C11—C12	110.3 (6)	H1A—C1—H1B	108.2
N4—C11—H11	108.3	N2—C2—C1^i^	108.5 (5)
C13—C11—H11	108.3	N2—C2—H2A	110.0
C12—C11—H11	108.3	C1^i^—C2—H2A	110.0
C6—C5—C3	119.3 (6)	N2—C2—H2B	110.0
C6—C5—H5A	107.5	C1^i^—C2—H2B	110.0
C3—C5—H5A	107.5	H2A—C2—H2B	108.4
C6—C5—H5B	107.5	H2WA—O2W—H2WB	109 (3)
C3—C5—H5B	107.5	H4WB—O4W—H4WA	109 (3)
H5A—C5—H5B	107.0	H3WA—O3W—H3WB	107 (3)
N3—C9—C10^ii^	107.7 (5)		
			
O1—W1—O4—Ni1	−113.9 (5)	N4^ii^—Ni2—N3—C14	−149.8 (5)
O2—W1—O4—Ni1	5.5 (6)	N4—Ni2—N3—C14	30.2 (5)
O3—W1—O4—Ni1	125.6 (5)	O1^ii^—Ni2—N3—C14	−59.9 (5)
O2—W1—O1—Ni2	73.4 (4)	O1—Ni2—N3—C14	120.1 (5)
O4—W1—O1—Ni2	−167.2 (3)	C1—N1—C6—C7	−53.1 (7)
O3—W1—O1—Ni2	−46.1 (4)	Ni1—N1—C6—C7	77.3 (6)
W1—O4—Ni1—N2	87.9 (5)	C1—N1—C6—C5	−175.7 (5)
W1—O4—Ni1—N2^i^	−92.1 (5)	Ni1—N1—C6—C5	−45.2 (7)
W1—O4—Ni1—N1^i^	2.5 (5)	C1—N1—C6—C8	67.5 (7)
W1—O4—Ni1—N1	−177.5 (5)	Ni1—N1—C6—C8	−162.1 (5)
W1—O1—Ni2—N3^ii^	117.7 (4)	C10—N4—C11—C13	−178.8 (5)
W1—O1—Ni2—N3	−62.3 (4)	Ni2—N4—C11—C13	59.7 (6)
W1—O1—Ni2—N4^ii^	−147.8 (4)	C10—N4—C11—C12	−56.2 (8)
W1—O1—Ni2—N4	32.2 (4)	Ni2—N4—C11—C12	−177.7 (5)
N2—Ni1—N1—C1	167.3 (4)	N1—C6—C5—C3	67.8 (7)
N2^i^—Ni1—N1—C1	−12.7 (4)	C7—C6—C5—C3	−54.3 (8)
O4—Ni1—N1—C1	76.4 (4)	C8—C6—C5—C3	−173.6 (6)
O4^i^—Ni1—N1—C1	−103.6 (4)	C14—N3—C9—C10^ii^	−179.4 (5)
N2—Ni1—N1—C6	32.0 (5)	Ni2—N3—C9—C10^ii^	43.7 (6)
N2^i^—Ni1—N1—C6	−148.0 (5)	C11—N4—C10—C9^ii^	−171.2 (5)
O4—Ni1—N1—C6	−58.9 (5)	Ni2—N4—C10—C9^ii^	−44.5 (6)
O4^i^—Ni1—N1—C6	121.1 (5)	C9—N3—C14—C13	−172.9 (5)
N1^i^—Ni1—N2—C2	16.8 (4)	Ni2—N3—C14—C13	−43.5 (7)
N1—Ni1—N2—C2	−163.2 (4)	C9—N3—C14—C16	−49.2 (8)
O4—Ni1—N2—C2	−68.4 (4)	Ni2—N3—C14—C16	80.2 (7)
O4^i^—Ni1—N2—C2	111.6 (4)	C9—N3—C14—C15	69.0 (7)
N1^i^—Ni1—N2—C3	143.2 (5)	Ni2—N3—C14—C15	−161.6 (5)
N1—Ni1—N2—C3	−36.8 (5)	N4—C11—C13—C14	−78.2 (8)
O4—Ni1—N2—C3	58.0 (4)	C12—C11—C13—C14	158.4 (6)
O4^i^—Ni1—N2—C3	−122.0 (4)	N3—C14—C13—C11	66.9 (8)
N3^ii^—Ni2—N4—C10	16.1 (4)	C16—C14—C13—C11	−57.8 (8)
N3—Ni2—N4—C10	−163.9 (4)	C15—C14—C13—C11	−174.6 (6)
O1^ii^—Ni2—N4—C10	−70.1 (4)	C2—N2—C3—C4	−56.5 (8)
O1—Ni2—N4—C10	109.9 (4)	Ni1—N2—C3—C4	−178.5 (5)
N3^ii^—Ni2—N4—C11	143.2 (5)	C2—N2—C3—C5	−179.3 (5)
N3—Ni2—N4—C11	−36.8 (5)	Ni1—N2—C3—C5	58.7 (7)
O1^ii^—Ni2—N4—C11	57.1 (4)	C6—C5—C3—N2	−77.9 (8)
O1—Ni2—N4—C11	−122.9 (4)	C6—C5—C3—C4	157.9 (6)
N4^ii^—Ni2—N3—C9	−15.4 (4)	C6—N1—C1—C2^i^	178.6 (5)
N4—Ni2—N3—C9	164.6 (4)	Ni1—N1—C1—C2^i^	40.5 (6)
O1^ii^—Ni2—N3—C9	74.5 (4)	C3—N2—C2—C1^i^	−170.8 (6)
O1—Ni2—N3—C9	−105.5 (4)	Ni1—N2—C2—C1^i^	−43.5 (6)

Symmetry codes: (i) −*x*+2, −*y*+2, −*z*+2; (ii) −*x*+2, −*y*+2, −*z*+1.

### Hydrogen-bond geometry (Å, º)

**Table:**

*D*—H···*A*	*D*—H	H···*A*	*D*···*A*	*D*—H···*A*
N1—H1*C*···O2^i^	0.93	2.32	3.253 (7)	180
N2—H2*C*···O4*W*^i^	0.93	2.21	3.040 (8)	149
O4*W*—H4*WB*···O3*W*	0.85 (2)	2.12 (5)	2.720 (8)	128 (6)
O4*W*—H4*WA*···O3	0.86 (2)	2.05 (3)	2.900 (7)	168 (6)
O2*W*—H2*WA*···O1*W*^iii^	0.85 (2)	1.94 (2)	2.790 (8)	175 (7)
O3*W*—H3*WA*···O2^iv^	0.87 (2)	2.01 (5)	2.784 (7)	148 (8)
O1*W*—H1*WA*···O2	0.86 (2)	1.95 (2)	2.801 (7)	172 (8)
O2*W*—H2*WB*···O3	0.86 (2)	1.99 (3)	2.811 (7)	160 (8)
O3*W*—H3*WB*···O2*W*	0.86 (2)	2.08 (4)	2.834 (8)	145 (6)
O1*W*—H1*WB*···O2*W*	0.85 (2)	2.10 (4)	2.895 (9)	157 (7)
C16—H16*A*···O1^ii^	0.98	2.40	3.241 (9)	144

Symmetry codes: (i) −*x*+2, −*y*+2, −*z*+2; (ii) −*x*+2, −*y*+2, −*z*+1; (iii) −*x*+1, −*y*+1, −*z*+1; (iv) *x*−1, *y*, *z*.
